# Association of A1AT genetic polymorphism and NSCLC: a case- control study in Egyptian population

**DOI:** 10.1186/s12920-023-01608-6

**Published:** 2023-07-27

**Authors:** Aliaa N. El-Dawa, Afaf M. ElSaid, Sherif Refaat, Omali Y. El-khawaga

**Affiliations:** 1 Biochemistry Division, Chemistry Department, Faculty of Science, Mansoura University, Mansoura, 35516 Egypt; 2 Genetic unit, Department of Pediatrics, Faculty of Medicine, Mansoura University, 35516 Mansoura, Egypt; 3 Lecturer of Medical Oncology, Faculty of Medicine, Mansoura University, Mansoura, Egypt

**Keywords:** Lung cancer, A1ATD, Biomarker, Genetics

## Abstract

**Background:**

Lung cancer mortality is higher than other forms of cancer. Genetic tendencies in cancer patients have long been known. Given the link between A1ATD and numerous lung disorders, it is worth investigating if this genetic trait is linked to a higher risk of developing LC, as the lung is the most afflicted organ in individuals with severe A1ATD. This study is intended to investigate the possible association between AAT rs17580 and rs8004738 gene polymorphisms and susceptibility to non-small cell lung cancer for early prediction in Egyptians.

**Methods:**

A case–control study was performed on 124 NSCLC cases and 124 healthy controls from 2021 to 2022 in the oncology center of Mansoura University. Peripheral blood was used to obtain genomic DNA. ARMS-PCR was used to genotype SNPs and other chemical parameters. Windows SPSS Statistics was used to review, encode, and tabulate the acquired data.

**Results:**

A molecular study for A1AT rs17580 and rs8004738 genotypes showed that NSCLC cases were significantly associated with a higher proportion of mutant S (T) and mutant Z (A) alleles (*p* = 0.042, 0.041, respectively). Different A1AT genotypes (MS, MZ, SS, SZ, and ZZ) showed no significant association with NSCLC or NLR.

**Conclusion:**

S and Z alleles might have significant impacts on NSCLC risk and can be useful for detecting and protecting individuals who may be vulnerable to carcinogens. Further research with larger sample sizes is needed to confirm the current findings.

**Supplementary Information:**

The online version contains supplementary material available at 10.1186/s12920-023-01608-6.

## Background

Cancer remains a primary public health problem. In 2020, there were 19.300.000 new cancer cases and 10.000.000 cancer-related deaths worldwide [1]. The most common cause of cancer death remains by far lung cancer (more than 1.800.000 deaths), followed by liver cancer (0.830.000) and stomach cancer (0.770.000) [1]. Lung cancer mortality is higher than that of other forms of cancer, often because of a late-stage diagnosis. Almost half of those with lung cancer (46%) are diagnosed after the cancer has already metastasized to other areas of the body, making it more difficult to treat [2]. Although lung cancer treatments have improved in recent years, early detection of this fatal disease remains a challenge. As a result, identifying specific genetic mutations associated with NSCLC is critical because they can help predict the disease at an early stage, increase the survivor rate, and help individualize treatments for this patient population. α-1antitrypsin (A1AT) is indeed an acute-phase glycoprotein that is primarily generated (by 80%) inside the human liver. After albumin and immunoglobulins, it is the most abundant blood protein. However, this protein can be expressed by various cell types [3, 4]. In general, the biological role of A1AT seems to be one of maintaining homeostasis and improving tissue repair and regeneration [5]. Its primary physiological role is to protect the lungs' matrix proteins (particularly elastin) against the proteolytic effects of proteases (particularly proteinase 3 and neutrophil elastase) generated by dying and activated neutrophils, as well as serine proteases secreted by invading bacteria. Alpha-1 antitrypsin deficiency (A1ATD) raises neutrophil elastase activity in the lungs, facilitating tissue degradation and causing emphysema [6].

A1ATD is primarily caused by mutations in the SERPINA1 gene, which result in either the creation of a misfolded protein that might lead to its retention inside the endoplasmic reticulum and lower serum levels, or perhaps the complete lack of a functional protein (null phenotype). SERPINA1 alleles are referred to as Pi* (protease inhibitor*), a term coined by Fagerhol and Laurell in 1967 that remains widely used today [7, 8]. Approximately 500 variants of the SERPINA1 gene are known to date [6]. More than 90 codominant alleles at the protease inhibitor locus have been identified [9]. The alphabetical abbreviations assigned to the different allelic variants denote their position in a pH gradient by isoelectric focusing. Leading researchers in this area designated these proteins as M (medium) for those with medium velocity, F (fast) for those with fast migration, and S (slow) for those with slow migration [10]. The wild type of functioning allele is PI*M, and the two most prevalent deficiency alleles are PI*Z rs28929474 (Glu342Lys) within exon 5, which is a substitution of guanine (G) for adenine (A), while glutamic acid (Glu) is replaced by lysine (Lys) in proteins. In addition, PI*S rs17580 (Glu 264 Val), which is present in exon 3 of the gene, where adenine (A) is substituted by thymine (T). At the protein level, valine (Val) is found instead of glutamic acid (Glu) [10], which together produce proteins with conformational changes that form stable polymers retained within hepatocytes without being secreted into the bloodstream and secondarily cause a decreased concentration of A1ATin blood and tissues [11], accounting for 95% of all cases of A1ATD [12].

The relationship between A1ATD and lung [13], breast [14], liver, or colorectal cancer [15] has been studied. Lung cancer, which has two primary types based on the histological size of cancer cells underneath the microscope: non-small cell (NSCLC) and small cell (SCLC), which account for 85% and 15% of patients, respectively, seems to have a high mortality rate in developed nations. Given the link between A1ATD and numerous lung disorders, it is worth investigating if this genetic trait is linked to a higher risk of developing LC, as the lung is the most afflicted organ in individuals with severe A1ATD [16]. Furthermore, various studies have been carried out to investigate the link between A1ATD and LC, with conflicting findings. So, the primary objective of this research was to examine the scientific data on the association between A1ATD PI*Z rs28929474 and PI*S genotypes with the probability of developing NSCLC in Egyptian patients. Secondary goals were to examine the impact of A1ATD on the histological subtype of NSLC and the neutrophil-lymph ratio (NLR) systemic inflammation response.

## Methods

This study is a case–control study performed on 124 histologically confirmed NSCLC cases under treatment or within diagnosis admitted to the Mansoura University Oncology Center from 2021 to 2022, together with 124 healthy age- and sex-matched individuals. Patients in the study were defined by the WHO, which has classified NSCLC into three major types: adenocarcinoma, squamous cell carcinoma, and large cell carcinoma [16]. Matched healthy controls would be recruited according to the absence of both clinical manifestations and a family history suggesting NSCLC cancer. Participants were excluded from other intestinal diseases or other parts of the original tumor. No one in the control group was a smoker or had a history of any interfering disease or chronic use of any drugs. There was only a minimal risk of local infection at the location of blood sampling, and full antiseptic measures were taken to avoid this risk. No unexpected risks appeared during the study. Waste products were burned in a combustion chamber by incineration process. The Ethics Board of Mansoura University IRB gave approval: MS.21.10.1721, date: 13/12/2021. A written informed consent was obtained from all caregivers of patients and control volunteers. The patients' confidentiality was maintained by assigning a code number to each of them. The results of this study could be published without revealing any information concerning the subjects' identities. All patients in this study underwent a comprehensive clinical examination, which included imaging tests using x-rays, magnetic fields, sound waves, or radioactive substances. TNM and stage, tumor histopathology, and grade are investigated in hospitals. In the laboratory, hematological parameters tests, including WBCs, neutrophils, and lymphocyte count, and molecular examinations for genetic changes were carried out.

### Blood sampling

Samples were gathered by taking 3 ml of blood from all patients and controls on sterile ethylene diamine tetra acetic acid (EDTA) for analysis of hematological parameters and A1AT gene polymorphisms. All samples were obtained and stored at -20°c. Prior to the procedure, they had been left at room temperature to be used in DNA extraction. then it was analyzed by PCR technique, followed by gel electrophoresis to detect gene polymorphisms.

### Genomic DNA Extraction

From a sample of 2 mL of peripheral blood in EDTA tubes, leukocytes were separated, and genomic DNA was obtained using the standard protocols of the commercial Easy Pure® Genomic DNA Purification Kit (Transe GEN Easy Pure® Genomic DNA Kit Cat. No. EE121-01). The DNA was quantified by ultraviolet light absorption spectrophotometry at 260 nm, protein contamination was determined at 280 nm, and a contaminant-free sample was considered when the ratio 260/280 was between 1.7 and 2.0. Each sample was adjusted to 25 ng/µL for subsequent genotyping.

### ARMS PCR for A1ATGENOTYPE

The A1AT genotype for S and Z alleles was assessed using a sequence specific primer (SSP)-based primer technique. Primers were developed using the Pi S and Pi Z gene sequences, GenBank sequence K02212 for the S variant and J02619 for the Z variant [17]. Two primers were designed for each allele. The primers produced amplicons of 221 base pairs for the S region and amplicons of 288 base pairs for the Z region Table 1.

**Table 1:** primer sequences used for PiZ and PiS AAT genotypes

**Mutation**	Primer sequence	Size (bp)
**Pi Z allele rs8004738**	Sense Z: 5´GCTGTGCTGACCATCGACA 3´	288 bp
	Anti-sense Z/ non-Z:5´ CCAGGGATTTACAGATCACATGC-3´	
	Sense non-Z:5´-GCTGTGCTGACCATCGACG-3´	288 bp
	Anti-sense Z/ non-Z:5´-CCAGGGATTTACAGATCACATGC-3´	
**Pi S allele rs17580**	Anti-sense S: 5´-ATGATATCGTGGGTGAGTTCATTTA-3´	221 bp
	Sense S/ non-S 5´-GAAGTCAAGGACACCGAGGA- 3´	
	Anti-sense non-S:5´-ATGATATCGTGGGTGAGTTCATTTT-3´	221 bp
	Sense S/ non-S 5´-GAAGTCAAGGACACCGAGGA- 3´	

### Primer-PCR program

**Fig. 1:**
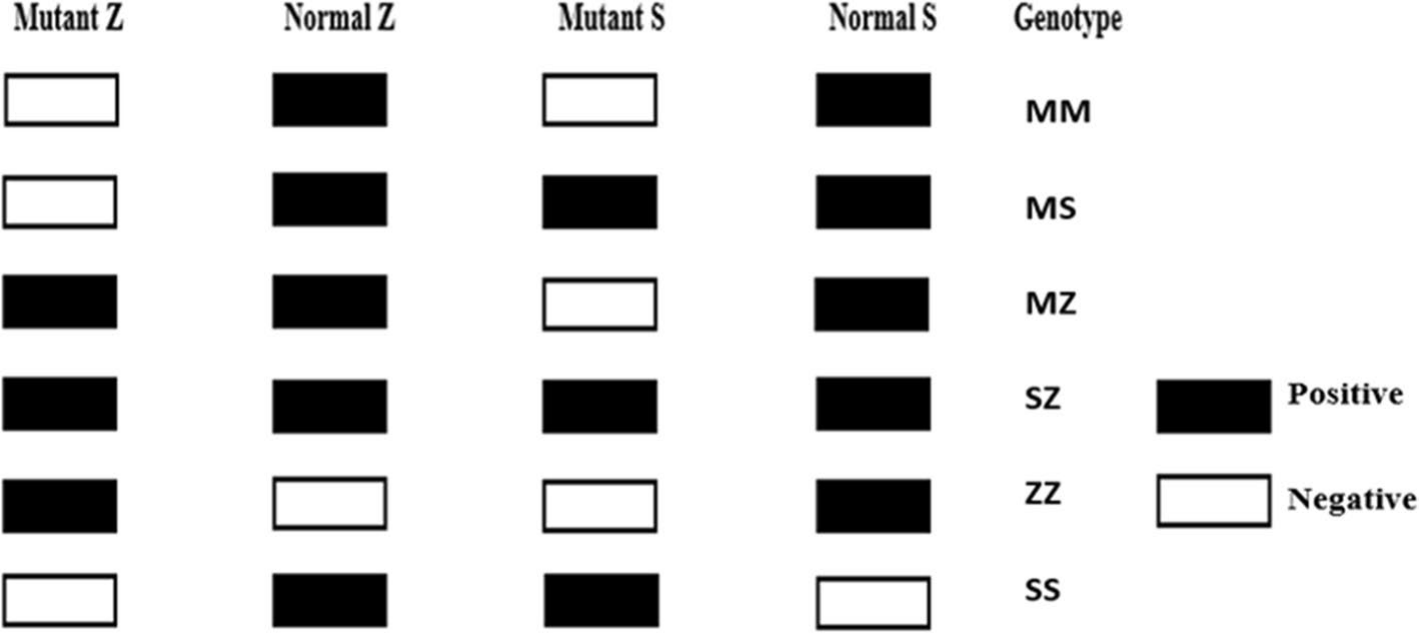
Schematic diagram of the possible results of ARMS-PCR of AAT genotype

Samples were amplified using the allele-specific primers at a concentration of 10 pmol/µl. Each tube contained two primers: 4 µl of each primer, 4 µl of the extracted DNA, and 12 µl of the Master Mix (COSMO PCR RED Master Mix (W10203001)), so the whole volume of the reaction was 24 µl. Samples were amplified using T professional thermocycler (Biometra, Germany). The PCR assay conditions were: initial denaturation at 96 °C, 2 min for 1 cycle; 30 cycles including denaturation at 96 °C, 25 s; annealing at 64.5 °C, 60 s; extension at 72 °C, 60 s; final extension at 72 °C, 2 min for 1 cycle; and then soaking at 4 °C. The PCR products were analyzed by electrophoresis on a 2.5% agarose gel containing 0.5 µg/mL of ethidium bromide and visualized by transillumination with ultraviolet light. A photograph was then taken. The results were interpreted into one of six groups, as shown in Fig. 1.

### Statistical analysis

Statistical Programming for Social Science (SPSS) Version 25.0 (IBM Corp., 2017. Windows SPSS Statistics, Version 25.0. Armonk, NY: IBM Corp.) was used to revise, encode, and tabulate the obtained data. Pearson's Chi-square test was used to examine the genotype distributions of mutations between patients and controls. 95% confidence interval and odd ratio (OR) conferred by potential correlations regarding the A1AT gene polymorphisms with the risk and progression of NSCLC. The probability level (P) of less than 0.05 was defined as a criterion of significance.

## Results

The current study represents a case–control comparative study of 124 unrelated patients with NSCLC and 124 control volunteers. The Hardy–Weinberg equation revealed that all studied genotypes in the control group as well as in NSCLC cases were in HW equilibrium, as no significant differences were found between observed and expected counts in each group. The research results include selected data from all evaluated parameters in control volunteers compared to NSCLC patients. Patients included 74 males, representing the majority of cases, and 50 females. The patients ages ranged with a (mean ± SD) of (56.0 ± 11.5) years. The control volunteers were 124 healthy individuals, including 78 males and 46 females. The controls` ages ranged with a (mean ± SD) of (55.4 ± 11.0) The two groups appeared to be adequately homogenous concerning age (*P* = 0.272) and gender (*P* = 0.602) distribution. In the present study, the hematological parameters such as white blood cell counts (WBCs) and neutrophil lymphocyte ratios (NLRs) in blood were shown in Table 2. The current results showed that NSCLC patients had higher WBCs, neutrophils, and NLR and lower lymphocytes, particularly in comparison to the healthy group (*p* ≤ 0.001 for each).

**Table 2:** comparison on different laboratory parameters among studied groups

	Control *n = *124			NSCLC *n = *124			*P*
	**Median**	**Minimum**	**Maximum**	**Median**	**Minimum**	**Maximum**	
**WBC**	6.00	3.80	10.00	9.88	3.80	36.40	< 0.001*
**Neutrophil**	56.00	25.00	62.30	65.90	32.00	89.77	< 0.001*
**Lymphocyte**	26.00	15.90	45.90	22.20	3.97	65.20	0.001*
**NLR**	2.17	0.87	3.29	3.00	0.49	22.29	< 0.001*

*NLR* Neutrophil to lymph ratio, *P* Probability, comparison between control and all NSCLC

^*^significant value

### Distribution of A1AT PiS rs17580 and PiZ rs8004738 gene polymorphisms in controls compared to NSCLC patients

**Fig. 2:**
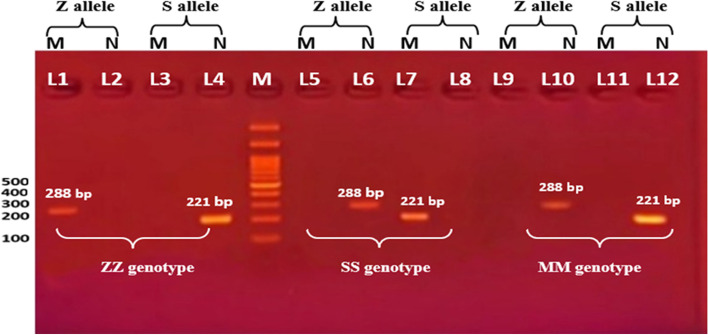
Shows gel electrophoresis of ARMS-PCR for AAT gene polymorphism. M indicates DNA ladder (100 bp). Each 4 lanes belong to only one participant. Lanes (1, 2, 5, 6, 9, and 10) represent Z allele bands. where band 288 bp appeared, Lanes (1, 5,9) represent Mutant Z alleles, lanes (2, 6, 10) represent normal Z allele. Lanes (3, 4, 7, 8, 11, and 12) represent S allele bands, where band appeared at 221 bp. lanes (3, 7, and 11) represent Mutant S allele. while Lanes (4,8, and 12) represent normal S allele. Lanes (1, 2, 3, 4) represent Mutant homozygote ZZ genotyping where mutant Z allele appeared at lane 1. and normal S allele at lane 4. Lanes (5, 6, 7, and 8) represent Mutant homozygote SS genotyping where mutant S allele appeared at lane 7, and normal Z allele at lane 6. Lanes (9, 10, 11, and 12), showed heterozygote MM genotyping where wild type of both Z and S allele appeared at lanes 10 and 12, respectively

**Fig. 3:**
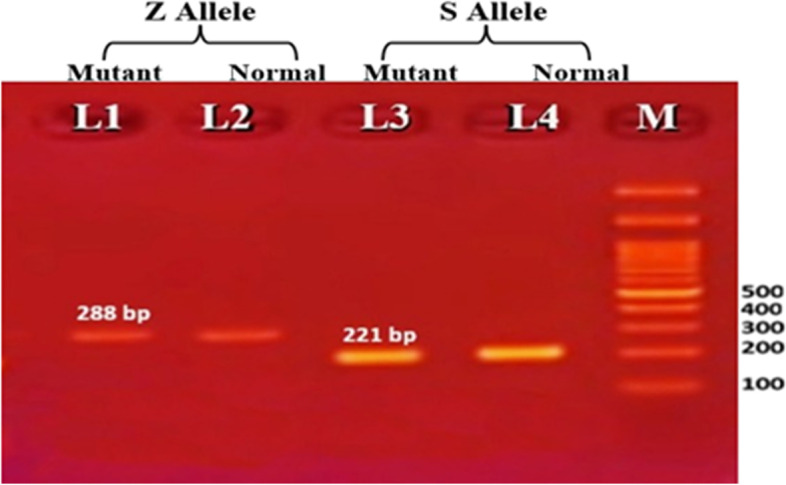
ARMS-PCR electrophoretic pattern of AAT gene polymorphism showing SZ genotype. M indicates DNA marker (100 bp). L1 and L2 represent the Z Allele. L3 and L4 represent the S allele. Amplicon (band) at 288 bp are present in both Lane 1 and 2 stand for heterozygosity of (Z Allele). Amplicon 221 bp in Lane 3 and 4 represent heterozygosity of (S Allele)

**Fig. 4:**
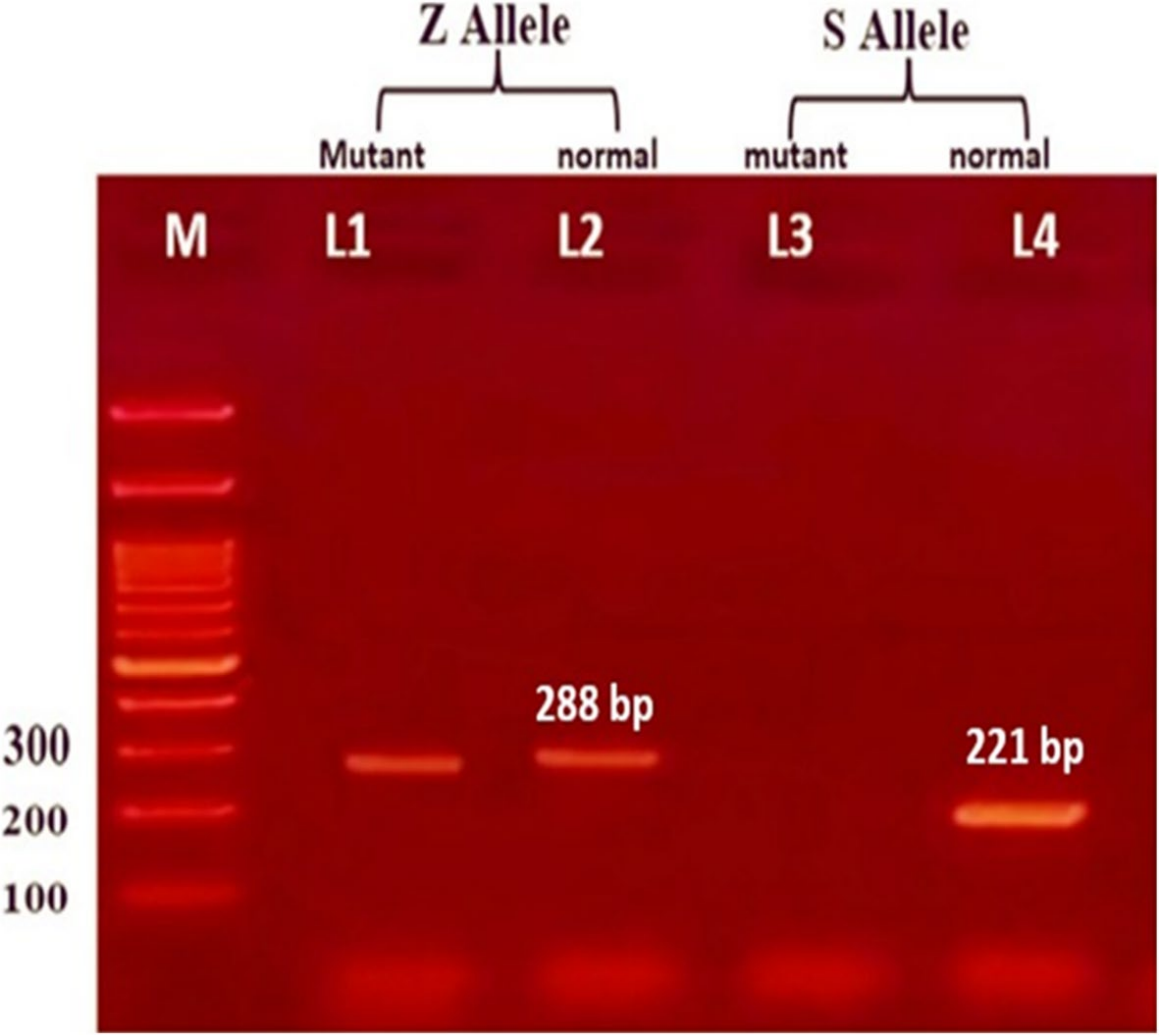
Shows gel electrophoresis of ARMS-PCR for AAT gene polymorphism. M indicate DNA ladder (100 bp) lanes (1,2) represent Z allele of normal and mutant, present at 288 bp, Lanes (3, 4) represent S allele of normal and mutant, where Lane (4) present at 221 bp of mutant, while wild S allele absent in this pattern indicated MZ genotyping

**Fig. 5:**
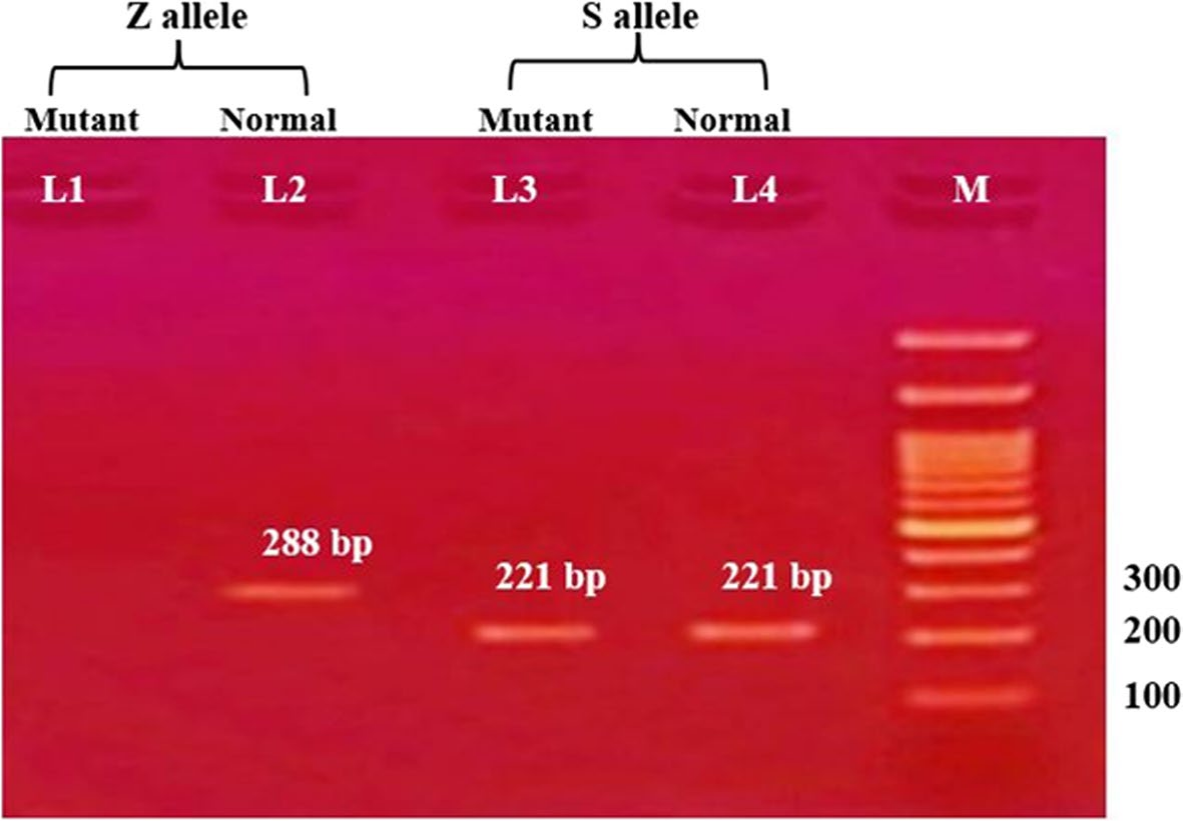
Shows gel electrophoresis of ARMS-PCR for AAT gene polymorphism. M indicate DNA ladder (100 bp). Lanes (3, 4) represent S allele of normal and mutant, appear at 221 bp. Lanes (1,2) represent Z allele of normal and mutant, lane (2) wild allele appears at 288 bp, while mutant Z allele absent in this pattern indicated MS genotyping

The different A1AT genotype distribution in patients and also control is illustrated in Table 3. Homozygous wild-type MM genotype was higher in control (96%) compared to NSCLC patients (84%). Heterozygous MS was more common in patients but did not reach statistical significance (*P* = 0.09). The remaining genotypes MZ, SS, SZ, and ZZ did not appear in healthy controls but only in patients. Considering the MM genotype is the reference normal genotype, no significant association was found between A1A2 genotypes and a risk of NSCLC susceptibility. However, NSCLC cases were significantly associated with a higher frequency of mutant Z allele (A) (*P* = 0.041) and Mutant S allele (T) (*P* = 0.042) when compared to the control (Figs. 2, 3, 4 and 5).

**Table 3:** A1A2 rs17580 and rs8004738 genotype and alleles frequency in NSCLC patients and healthy controls

A1AT			Control *n = *124		NSCLC *n = *124		p	OR	(95% CI)
			**n**	**%**	**n**	**%**			
**genotyping**	PiS and PiZ interpretation	**MM**	119	96.0	105	84.7	Reference		
		**MS**	5	4.0	11	8.9	0.090	**1**.774	(0.915–3.440)
		**MZ**	0	0.0	3	2.4	1	**-**	
		**SS**	0	0.0	3	2.4	1	**-**	
		**SZ**	0	0.0	1	0.8	1	**-**	
		**ZZ**	0	0.0	1	0.8	1	**-**	
**Alleles**	**rs8004738**	Mutant Z** (A)**	0	0.0	5	4.0	0.041*		
		Normal Z** (G)**	124	100	121	97.6	0.255		
	**rs17580**	Mutant S** (T)**	5	4.1	16	12.9	0.042*		
		Normal S** (A)**	119	96.9	121	97.6	0.255		

*OR* Odd ratio, *CI* Confidence interval, *P* Probability, P* significant value

### Association of A1AT gene polymorphism with other parameters in NSCLC patients

The criteria for selecting cases for proper DNA genotyping should depend on the histopathology and grade of the tumor tissue. Of the 124 patients selected, 98 (79.0%) were adenocarcinomas, 12 (9.7%) were determined to be large cell carcinomas, 8 (6.5%) were squamous cell carcinomas, and 6 (4.8%) were characterized as other sub-types of NSCLC. According to the tumor grade, 77 (64.2%) of patients were in the third grade, 41 (34.2%) of patients were in the second grade, and only 2 (1.7%) patients were in the first grade. The lung cancer stages of the participating patients were clarified according to lung cancer TNM staging (8th edition) [18]

Patients were classified using a combined pathological system for tumor tissues into stages I, II, III, and IV. There were 94 cases (75.8%) in the IV stage and 25 patients (20.2%) in the III stage. We explored the correlation between numerous factors and the A1AT genotype and depicted it in Table 4. The relationship of A1AT Pi S rs17580 and Pi Z rs8004738 with TNM staging in all NSCLC patients indicated no significant association among them (*P* = 0.615). Two-sided χ tests revealed no significant association between histology subtype (P = 0.27) or grade (*P* = 0.448) regarding A1A2 MM, MS, MZ, SS, ZZ, or SZ genotyping. There was no significant association between A1AT PiS and PiZ and blood laboratory parameter in all NSCLC cases (*P* > 0.05). Also, no significant association was found regarding A1AT and gender among all studied NSCLC patients (*P* = 0.69) Table 5.

**Table 4:** Association of A1ATgenotyping with laboratory parameters among all studied NSCLC cases

	MM		MS		MZ		SS		SZ		ZZ		*P*
	**M**edian	**Range**	Median	**Range**	Median	**Range**	Median	**Range**	Median	**Range**	Median	**Range**	
**WBCs**	9.8	(3.8 -36.4)	9.49	(5.59–20.2)	11.5	(11–12.2)	9.88	(6.9–10.9)	12.01	(12.0–12.01)	5.93	(5.93–5.93)	0.602
**Platelets**	293	(37.6 -542)	298	(118–510)	325	(221–364)	284	(106–374)	340	(340–340)	121	(121–121)	0.683
**Neutrophil**	66.1	(32 -89.8)	60.3	(44.2–83.2)	61.8	(55.7–80)	67.1	(52–69)	72.1	(72.1–72.1)	42.9	(42.9–42.9)	0.662
**Lymphocyte**	22	(3.97 -65.2)	22.5	(7.06–40)	27.3	(13.9–31.3)	22.3	(16.6–40.2)	18.9	(18.9–18.9)	46.2	(46.2–46.2)	0.664
**NLR**	3.01	(0.49 -22.2)	2.75	(1.22–11.7)	2.26	(1.7–5.76)	3.01	(1.29–4.16)	3.81	(3.81–3.81)	0.93	(0.93–0.93)	0.649

*NLR* Neutrophil to lymph ratio, P Probability, comparison between NSCLC cases

**Table 5:** Association of several variables with the A1AT different genotyping

	MM		MS		MZ		SS		SZ		ZZ		*P*
	**n**	**%**	**n**	**%**	**n**	**%**	**n**	**%**	**n**	**%**	**N**	**%**	
**Gender**													
**Males**	62	59.0	7	63.6	2	66.7	2	66.7	0	0.0	1	100.0	0.690
**Females**	43	41.0	4	36.4	1	33.3	1	33.3	1	100.0	0	0.0	
**Pathology**													
**adenocarcinoma**	85	81.0	7	63.6	2	66.7	3	100.0	1	100.0	0	0.0	0.207
**large cell carcinoma**	8	7.6	3	27.3	1	33.3	0	0.0	0	0.0	0	0.0	
**squamous cell**	6	5.7	1	9.1	0	0.0	0	0.0	0	0.0	1	100.0	
**Others**	6	5.7	0	0.0	0	0.0	0	0.0	0	0.0	0	0.0	
**Grade**													
**1**	1	1.0	1	9.1	0	0.0	0	0.0	0	0.0	0	0.0	0.448
**2**	35	34.7	3	27.3	1	33.3	2	66.7	0	0.0	0	0.0	
**3**	65	64.4	7	63.6	2	66.7	1	33.3	1	100.0	1	100.0	
**Stage**													
**I**	1	1.0	1	9.1	0	0.0	0	0.0	0	0.0	0	0.0	0.615
**II**	3	2.9	0	0.0	0	0.0	0	0.0	0	0.0	0	0.0	
**III**	21	20.0	3	27.3	1	33.3	0	0.0	0	0.0	0	0.0	
**IV**	80	76.2	7	63.6	2	66.7	3	100.0	1	100.0	1	100.0	

P, comparison of MM, MS, MZ, SZ, ZZ, SS genotypes, *P*-value determined by the χ squared test

## Discussion

Human A1AT is encoded by the SERPINA1 gene, which is found on the 14th chromosome at q31-q32. The A1AT gene has two alleles that are passed down through recessive autosomal codominant Mendelian inheritance [19]. A1ATD results from variations in the SERPINA1 gene that result in either low serum levels or a complete absence of protein output. SERPINA1 mutations have been described in over 150 different ways. The great majority of genotypes observed in clinical practice are M, Z, and S allele combinations, that is, Pi*MM represents the most common, non‑disease‑causing and is present in 85%–95% of people. While Pi*MS, Pi*SS, Pi*MZ, Pi*SZ, and Pi*ZZ are five deficiency genotypes present in almost all the disease-associated SERPINA1 mutations, since the description of AADT in the early 60th, it has become a major etiological factor for lung and liver disease. Also, the link between A1ATD and lung cancer has been investigated [13]. Therefore, it was necessary to study the most important genetic mutations affecting the lung within this gene and determine their relationship to the incidence of lung cancer. In the current study, we assessed the prevalence of A1AT rs17580 and rs8004738 genotypes among 124 advanced NSCLC patients and 124 healthy age- and sex-matched volunteers. The prevalence of Pi*MS, Pi*SS, Pi*MZ, Pi*SZ, and Pi*ZZ deficiency genotypes did not cross the significance border. We can estimate that no higher risk of NSCLC was found among individuals who were homozygous or heterozygous carriers of the most frequent A1AT deficiency alleles (PI*S, PI*Z) as compared to carriers of the normal genotype (PI*MM). Similarly, no effect was observed for patient subjects by age group. A Serbian study showed the frequency of A1ATD heterozygotes (MS, MZ) in the group of all 186 patients with lung cancer was 5.9% vs. 3.7% in the healthy subjects [9], lower than our results that show that in all of the 124 NSCLC patients, the heterozygosity was 11.3% vs. 4.9% in healthy subjects. The results of comparative studies vary greatly between different races. Also, our hypothesis that A1ATD carrier phenotypes could be associated with different types of NSCLC was not quite confirmed. However, the overall prevalence of the disease-associated S mutant (T) allele was 12.9% (16 cases) instead of only 4.0% (5 cases) in healthy controls. While the NSCLC patient-related Z mutant (A) allele was 4.0% (5 subjects) and no mutation appeared in healthy persons, both S and Z mutant alleles were found to be a substantial and important predicting factor for NSCLC in contrast to healthy individuals. Our findings are consistent with a prior study that revealed A1ATD alleles, mostly S and Z, to be associated with a considerably higher lung cancer risk among A1ATD carriers in a case–control comparison of 1856 patients who had incident lung cancer versus 1585 community residents as controls [18].

In cancer patients, a systemic inflammatory response is increasingly recognized as a prognostic marker. Cancer-related inflammation is marked by the appearance of inflammatory cells as well as inflammatory mediators, as well as tissue remodeling and angiogenesis elements similar to those observed in chronic inflammatory illnesses or tissue healing [20]. Several studies, for example, have indicated that the neutrophil-lymph proportion has predictive significance in predicting the survival rate of patients with NSCLC [21, 22]. Because A1AT seems to be a protein of the acute phase, several studies have been conducted to study the association between the serpin A1 gene Pi*S and Pi*Z genotypes and other acute phase indicators of inflammation such as the NLR [23, 24]. In the present research, we analyzed serum NLR in 124 NSCLC patients and another 124 healthy volunteers. High neutrophil and low lymphocyte count showed a median NLR of 3.98 versus 2.17 for patients and healthy controls, respectively, and showed significant NLR with NSCLC when compared to healthy volunteers (P ≤ 0.001). Surprisingly, we found no link between serum NLR and various A1AT genotypes in the tumor of the corresponding patients (*P* = 0.649). Similar observations were made in an important study published in 2019 at the German Agency for Lung Research, in which tumor and surrounding non-tumor tissues from the lung of 351 NSCLC patients were analyzed for SERPINA1 expression. Their findings show that the SERPINA1 gene does not only reflect inflammatory responses associated with cancer formation but also plays an important role in NSCLC pathogenesis [25]. Different studies have been recognized with the principal aim of analyzing the possible increase in risk of specific NSCLC subtypes associated with A1ATD genotypes. One example is the findings of Yang et al., who performed case–control research inside the United States and reported a 70% increase in the probability of LC related to A1AT-deficient genotypes, with adenocarcinoma and squamous-cell carcinoma lineages predominating [13]. Topic et al. discovered that having a deficient genotype of A1AT enhanced the incidence of squamous cell cancer in a case–control study on the Serbian population. This association, however, was not detected in adenocarcinoma or large cell carcinoma histology [9]. A different finding was observed in the present research, as there is no correlation among A1ATD genotypes and NSCLC histopathology subtypes (*P* = 0.207). That discrepancy could be explained by the assumption that this investigation discovered a higher prevalence of ADC (79.0%) and a lower incidence of SQC (4.8%) compared to the universal proportions.

## Conclusion

Our findings suggest that S and Z alleles have a potentially substantial influence on NSCLC risk, as well as the potential advantage of identifying and safeguarding these individuals who may be exposed to carcinogens. Despite the fact that the number of A1ATD carriers in the community is less than the proportion contributing to lung cancer risk, larger research might validate the possibility of putting this mutation as a genetics diagnostic among the top for important gene impacts on the risk of NSCLC.

## Supplementary Information

**Additional file 1.**

## Data Availability

The datasets generated and analyzed during the current study are available from the corresponding author on reasonable request.

## Abbreviations

AAT: Alpha-1 antitrypsin
AATD: Alpha-1 antitrypsin deficiency
ARMS: Amplification-refractory mutation system
NSCLC: Non–small- cell lung carcinoma
SERPINA1: Serpin peptidase inhibitor, clade A, member 1
