# Supplementary material for: Association of A1AT genetic polymorphism and NSCLC: a case- control study in Egyptian population
Source: BMC Med Genomics. 2023 Jul 27;16:173. doi: 10.1186/s12920-023-01608-6 (PMC10373285; doi:10.1186/s12920-023-01608-6)

**Figure and its original one:**

**Figure 3 and figure 4:**


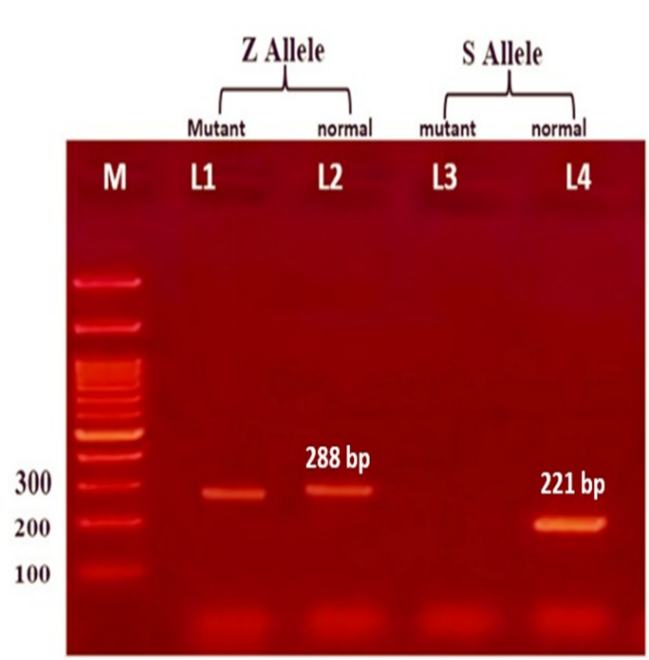
**
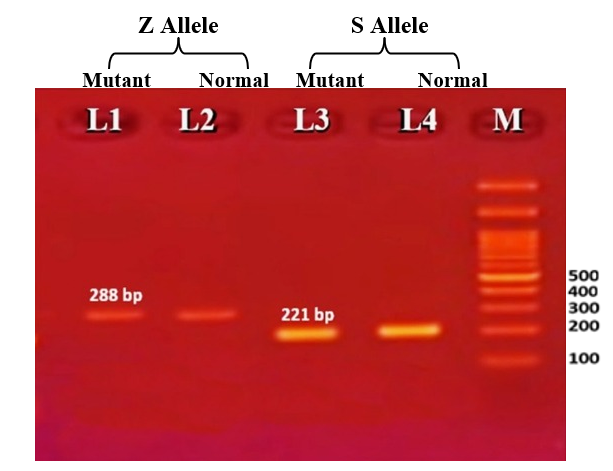
**

**The original gel figure of figure 3 and figure 4 (as both genotype in the same gel):**


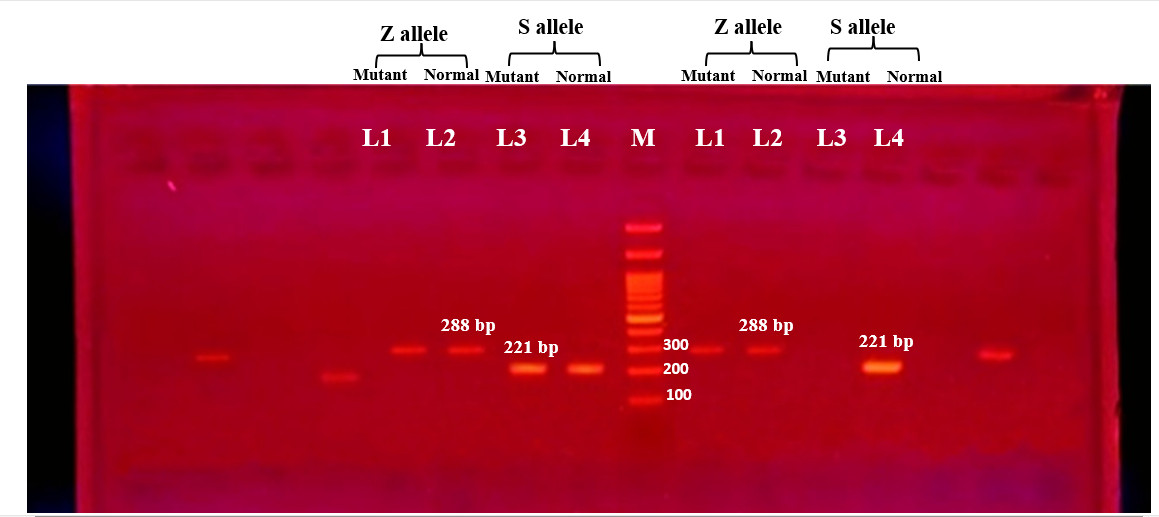


**
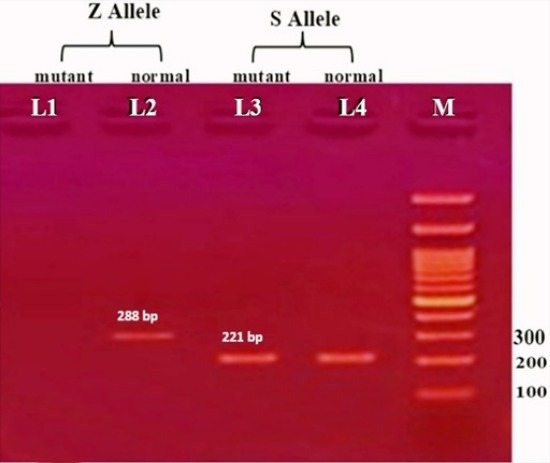
Figure 5:**

**The original figure 5 gel:**


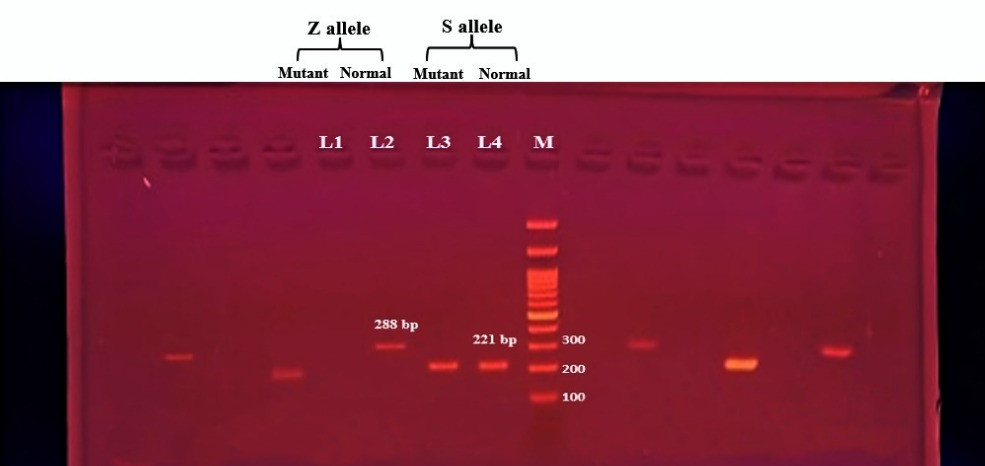

Supplement: Supplementary file 1 — Additional file 1. [file 12920_2023_1608_MOESM1_ESM.docx]
